# Visual accelerated and olfactory decelerated responses during multimodal learning in honeybees

**DOI:** 10.3389/fphys.2023.1257465

**Published:** 2023-10-20

**Authors:** Martin Strube-Bloss, Patrick Günzel, Carmen A. Nebauer, Johannes Spaethe

**Affiliations:** ^1^ Department of Biological Cybernetics, Faculty of Biology, Bielefeld University, Bielefeld, Germany; ^2^ Department of Plant-Insect-Interaction, Life Science Systems, Technical University of Munich, Freising, Germany; ^3^ Behavioral Physiology and Sociobiology (Zoology II), Biocenter, University of Wuerzburg, Wuerzburg, Germany

**Keywords:** multimodal integration, olfaction, vision, classical conditioning, honeybees, response time, M17-recordings

## Abstract

To obtain accurate information about the outside world and to make appropriate decisions, animals often combine information from different sensory pathways to form a comprehensive representation of their environment. This process of multimodal integration is poorly understood, but it is common view that the single elements of a multimodal stimulus influence each other’s perception by enhancing or suppressing their neural representation. The neuronal level of interference might be manifold, for instance, an enhancement might increase, whereas suppression might decrease behavioural response times. In order to investigate this in an insect behavioural model, the Western honeybee, we trained individual bees to associate a sugar reward with an odour, a light, or a combined olfactory-visual stimulus, using the proboscis extension response (PER). We precisely monitored the PER latency (the time between stimulus onset and the first response of the proboscis) by recording the muscle M17, which innervates the proboscis. We found that odours evoked a fast response, whereas visual stimuli elicited a delayed PER. Interestingly, the combined stimulus showed a response time in between the unimodal stimuli, suggesting that olfactory-visual integration accelerates visual responses but decelerates the olfactory response time.

## 1 Introduction

Multisensory integration is of central importance for perception as the environment usually comprises a potpourri of different sensory modalities. In fact, for almost all behaviours, the neural network must combine and define the relevance of different stimuli to obtain accurate information about the outside world and to make efficient decisions. This behaviours and their underlying neural mechanisms can be studied relatively straight forward in different insect species. Besides receiving stimuli at the insect antenna, which in itself represents an actively movable, multisensory organ (Dürr et al., 2022), beetles and ants integrate antennal information with visual stimuli to enhance their performance during short and long distance navigation (Dacke et al., 2019; Buehlmann et al., 2020a; Buehlmann et al., 2020b).

Flower blossoms provide visual and olfactory cues, which are used by naïve and experienced honeybees alike for proper flower detection during foraging (Giurfa et al., 1995; Raguso, 2004). Although most studies in honeybees investigated either the visual or the olfactory modality, a honeybee is probably experiencing a multimodal olfactory-visual construct of “smelling colours” and “colourful smells” when flying over a flower meadow (*reviewed by:* (Leonard and Masek, 2014)). Both single modalities - olfaction and vision - have been used in classical conditioning experiments by means of the proboscis extension response (PER) (olfactory stimuli *see review by:* (Matsumoto et al., 2012), visual stimuli (Hori et al., 2006; Niggebrügge et al., 2009; Dobrin and Fahrbach, 2012; Lichtenstein et al., 2018)). However, recent studies have shown that also combined stimuli (e.g., olfactory and visual) can be learned in classical conditioning experiments in honeybees (Mansur et al., 2018; Becker et al., 2019) and bumblebees (Riveros et al., 2020; Riveros, 2023).

Compared to a unimodal olfactory or visual reward association, the complexity of a multimodal olfactory-visual reward association might enhance or suppress the perception of the single modalities (elements) [examples of that ambivalence in multimodal integration can be found in (Calvert et al., 2004)]. To investigate whether this also applies to bees, we trained honeybees in a series of experiments to olfactory (experiment 1), visual (experiment 2) and olfactory-visual compound stimuli (experiment 3). From a behavioural perspective, a cross modal interaction during olfactory-visual integration might result in a prolonged neural computation time, since the brain must cope with parallel information channels, which must be compared and merged. This may affect the underlying neural processing of perception and further the decision time and would lead to a shift in the behavioural response latency.

A prerequisite of merging modalities is a neural convergence of the involved pathways at a higher order integration centre. In honeybees, unimodal projection neurons (PN) of both, the olfactory and the visual primary neuropils, innervate the calyces of the mushroom bodies (MB), representing the first neural level of olfactory-visual convergence (*reviewed by:* (Groh and Rössler, 2020)). Moreover, at this processing level both modalities converge with the reward pathway, which is facilitated by the ventral unpaired median neuron number one of the maxillary neuromere (VUMmx1), which broadly innervates the input region of the MB (Hammer, 1997). The output of the MB is mediated by a few hundred MB output neurons (MBON) with long-range centrifugal connections to the lateral horn, the protocerebral lobe and the antennal lobe (AL), as well as close-range connections to the MB calyx input (Rybak and Menzel, 1993; Strausfeld, 2002). Taken together, the morphological convergence suggests multimodal interactions between visual, olfactory and reward projections at the MB level.

Indeed, extracellular long-term recordings of MBONs recently revealed that a subpopulation of about 32% of MBONs were sensitive to both modalities, integrating olfactory and visual information. Other subpopulations responded to only visual (42%), or olfactory (9%), or showed no response (17%) to the tested stimuli (Strube-Bloss and Rössler, 2018). A similar distribution was suggested by a connectome study in *Drosophila*, with some MBONs predominantly assigned to code for non-olfactory modalities (Li et al., 2020). In unconditioned bees, maximal inter- and intra-stimulus separation in MBONs is reached at about 200 ms after stimulus onset (Strube-Bloss et al., 2011; Strube-Bloss and Rössler, 2018). Moreover, the MB output encodes behavioural decisions during sensory-motor transformation in untrained cockroaches (Arican et al., 2022). However, classical conditioning experiments in combination with MBON recordings in honeybees showed a recruitment of initially non-responding MBONs to encode the stimulus reward association (Strube-Bloss et al., 2011; Strube-Bloss et al., 2016). Most interestingly, the computation of the reward associated stimulus (CS+) was prolonged, reaching its maximum stimulus separation about 100 ms later compared to neutral stimuli (Strube-Bloss et al., 2011). It is therefore conceivable that a prolonged computation in the MB might affect the response time and, as a consequence, the timing of the PER.

Therefore, we monitored the first behavioural reaction during the conditioning of the PER by recording an electromyogram of the muscle M17, which is innervating the proboscis (Rehder, 1987; Smith and Menzel, 1989a). By quantifying the behavioural response latencies after classical odour (experiment 1) and light (experiment 2) conditioning, we were able to compare the modality-specific responses. We found that while odours evoked a fast PER, light stimuli evoked a delayed PER after reward association. Most strikingly, the PER latency for the olfactory-visual reward association (experiment 3) was in between. Thus, olfactory-visual compound association accelerates (enhances) light, but slows down (suppresses) odour induced response behaviour.

## 2 Materials and methods

### 2.1 Animals

Worker honeybees were collected from outdoor colonies in the afternoon before the training day, immobilized on ice, restrained into small cylindrical metal tubes, and fed *ad libitum* with 30% sugar-water solution. They were then kept overnight in a dark, humid chamber (25°C and 60% humidity). In the morning of the following day, these bees were tested for their motivational state by touching the antennae with a 50% sucrose solution. Only bees which exhibited a clear PER were chosen for the experiment. Since recording electrodes were inserted, only one bee was trained at a time.

### 2.2 Olfactory conditioning (experiment 1)

**FIGURE 1:**
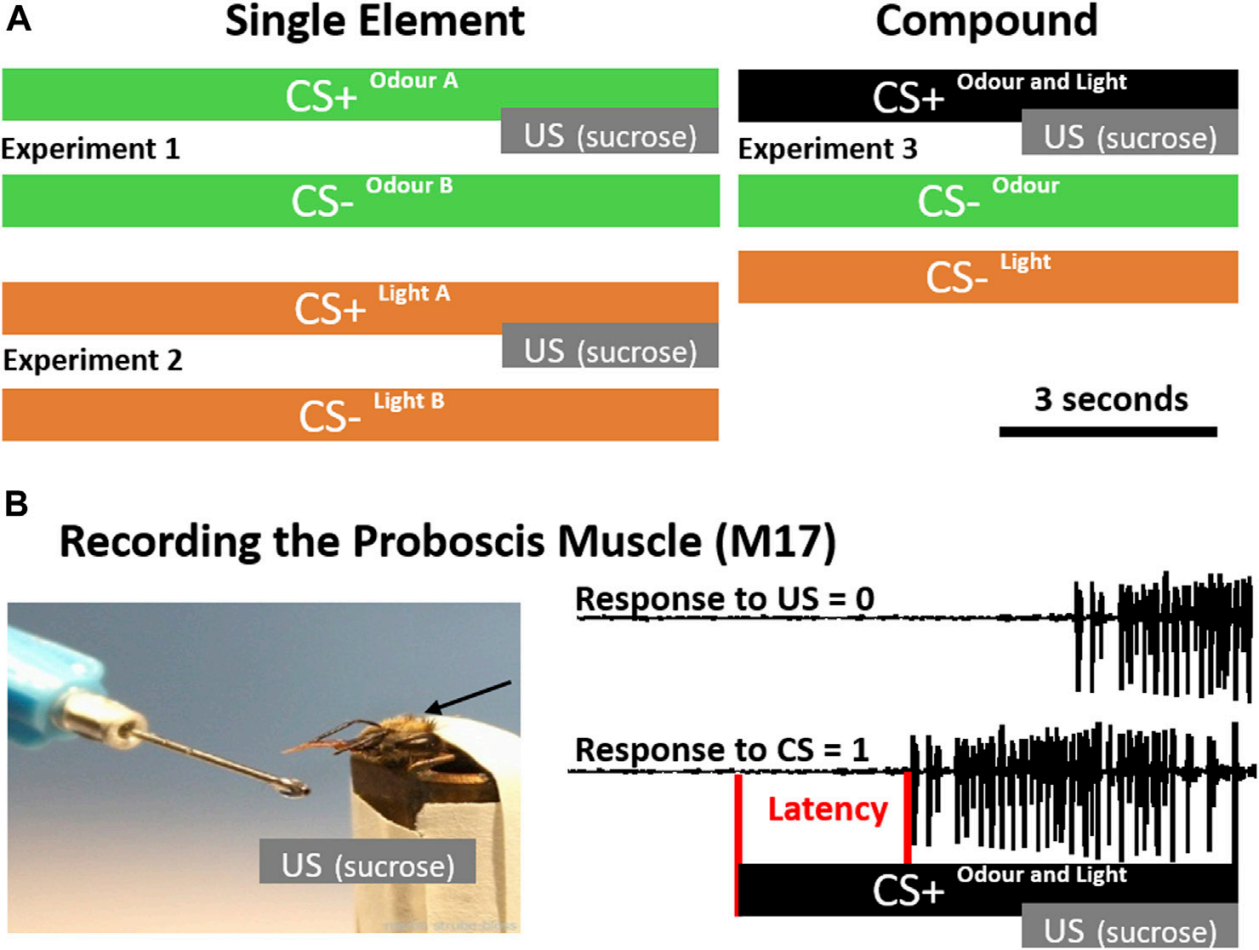
Conditioning Protocols and Latency Detection. **(A)** During single-element conditioning (left), either two odours (green, experiment 1) or two light stimuli (orange, experiment 2) were presented. In each experiment one of the stimuli was rewarded by sucrose (CS+) and the other one was unrewarded (CS-). During compound conditioning (right) the single elements odour and light were presented unrewarded (CS-), however, if presented together in a compound (black), they were rewarded (experiment 3). **(B)** To precisely monitor the proboscis extension response, we recorded the muscle (M17) which innervates the proboscis. During the acquisition phase, a positive response was counted if M17 activity starts after CS onset before the unconditioned stimulus (US; sucrose) was presented (CS = 1). If M17 activity started in response to the US, it was counted as not associated (US = 0). In addition the latency between stimulus onset and activation of the muscle (red) was analysed.

Animals were placed in front of an exhauster. Only one bee at a time was conditioned to differentiate between 1-Nonanol and 2-Hexanol. One odour was rewarded (CS+) and overlapped with an unconditioned stimulus (US; sucrose solution), the other one was unrewarded (CS-) without US (Experiment 1, Figure 1A). Eight bees received 1-Nonanol as CS+ and 2-Hexanol as CS-, whereas and eight bees were trained contrariwise. As both combinations were learned equally well, data were combined (Supplementary Figure S1). For olfactory stimulation, a Syntech CS-55 (Ockenfels Syntech GmbH, Kirchzarten, Germany) was used, generating a continuous air flow (1.0 L/min) in which the odour stimulus was injected by switching between an empty pipette (permanent stimulus) and a pipette prepared with a filter paper (2 cm^2^) soaked with 5 µL of the pure odorant. Switching between the two chambers avoids pressure loss and minimised physical artefacts. CS+ and CS- were presented 10 times each, in a pseudo-randomised order. Stimulation lasted 10 s with an ITI of 10 min. The US lasted 3 s and overlapped with the last 3 seconds of the CS+ (Figure 1A).

### 2.3 Visual conditioning (experiment 2)

Animals were placed in a box (about 10 × 10 cm) which could be illuminated from above. Only one bee at a time was conditioned to differentiate between blue (435 nm) and green (488 nm) light, provided by a cold light lamp (Schott, KL1500 electronic) and monochromatic filters. One light stimulus was rewarded (CS+) and overlapped with an unconditioned stimulus (US; sucrose), the other one was unrewarded (CS-) without a US (Experiment 2, Figure 1A). Eight bees received green light as CS+ and blue light as CS-, whereas again eight bees were trained contrariwise. As both combinations were learned equally well, data were combined (Supplementary Figure S1). Stimulus duration was controlled using an electronical shutter driver (A Vincent Associates, UNIBLITZ Model VCM-D1, Rochester, NY, United States of America) and set to 10 s. The US lasted 3 s and overlapped with the last 3 seconds of light stimulation (Figure 1A). CS+ and CS- were presented 10 times each, in a pseudorandomized order with an ITI of 10 min. The experimental setup remained unchanged during both visual and olfactory conditioning (i.e., the lamp was kept above during olfactory conditioning and the pipette remained in front of the animal during visual conditioning) in order to keep a constant environment.

### 2.4 Olfactory-visual compound conditioning (experiment 3)

During compound conditioning we followed our recently established procedure (Becker et al., 2019). In short: two odours (Hexanal and Geraniol) were delivered as described above using a Syntech CS-55. To control and synchronise the light stimulation with the odour presentation, we used the TTL output of the Syntech CS-55. Two monochromatic LEDs (UV: 375 nm, intensity: 7.5×10^13^ photons cm^−2^ s^−1^; green: 525 nm, intensity: 3.93×10^13^ photons cm^−2^ s^−1^) were placed at the end of a transparent Plexiglass rod (diameter: 10 mm, length: 100 mm). Light transmission through the Plexiglass rod ensured diffusion before the light would reach the eye. Our custom build light device was positioned 3 cm above the bee’s head and orthogonal to the odour stimulation device (see: Becker et al., 2019). The experimenter could switch manually between the LEDs or set the light stimulus off, if an odour was presented alone. To present light alone, the stimulus pipette including the odour soaked filter paper was replaced by another empty pipette. To make sure that odour identity as well as light identity had no influence on the discrimination performance, each odour stimulus was combined with each light stimulus resulting in four different odour-light combinations. For each combination, 15 bees were trained to discriminate the unrewarded single elements light and odour (CS-) from the rewarded olfactory-visual compound stimulus (CS+). CS+ and CS- were presented 10 times each, in a pseudo-randomised fashion. Since there were 20 CS- trials (10 x odour, 10 x light), we shortened the experimental procedure to keep the motivation of the bees high by using an ITI of 5 minutes. Additionally, the CS+ and CS- presentation was shortened to 7 s, whereas the US presentation was same as before (Figure 1A). In all four combinations, bees learned to associate the olfactory-visual compound with the reward and to discriminate it from its single unrewarded elements (odour alone, light alone; Supplementary Figure S2).

### 2.5 Monitoring the M17 muscle

In order to monitor the M17 muscle, we recorded differentially from two isolated silver wires (diameter: 20 µm) inserted at the approximate attachment point of the M-17 muscles between the ocelli and the compound eyes (Rehder, 1987; Smith and Menzel, 1989a). One wire was positioned on the left, the other one on the right hemisphere. To reduce electrostatic noise and get better visual access to the bees ‘neck’, the head was shaved with a razor blade beforehand. A third wire served as ground electrode and was attached to the metal tube. Recording signals were sampled at 15 kHz using an A/D converter (CED Micro1401, Cambridge Electronic Design, Cambridge, United Kingdom), and visualised using Spike2 software (Cambridge Electronic Design, Cambridge, United Kingdom). In parallel, we recorded a TTL signal of the stimulation devices to precisely analyse response latencies (Figure 1B). Please note, that the latency distribution analysis in Figure 5 includes only trials, in which bees responded to the CS + stimulus. In trials with no response, we were not able to calculate a latency. Temporal parameters like stimulation time and inter-trial interval (ITI) were controlled using the computer programme “TimingProtocol“ (Lichtenstein et al., 2018), which provided precise acoustical cues to the experimenter.

### 2.6 Statistical analysis

**FIGURE 2:**
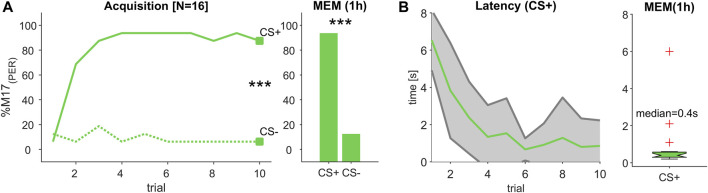
(Experiment 1): Decreasing response latency during odour learning. **(A)** The percentage of bees responding with proboscis extension measured by recording the M17 muscle (% M17 (PER); *y*-axis) over successive conditioning trial (*x*-axis) shows that bees reliably learned to differentiate between a rewarded (CS+) and an unrewarded (CS-) odour stimulus resulting in a stable memory (MEM), which was tested 1 hour after the last acquisition trial (trial 10 and MEM: Chi2-test; *p* < 0.001). **(B)** During acquisition, response latency decreased (green line, ±SD in grey). The median response time during memory test was ca. 400 m.

**FIGURE 3:**
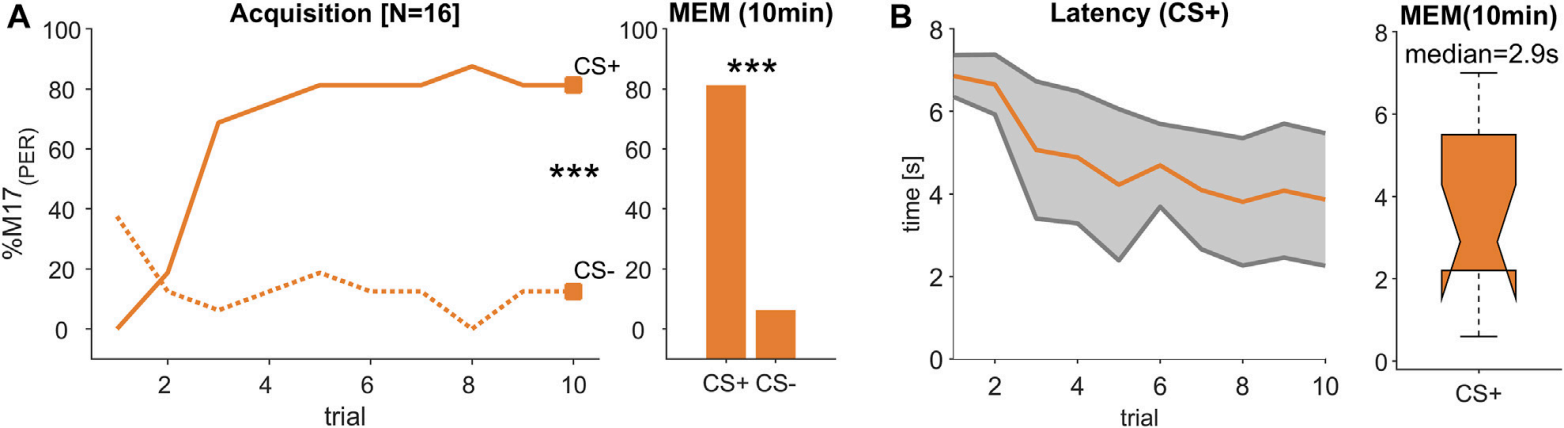
(Experiment 2): Decreasing response latency during visual learning. **(A)** The percentage of bees responding with proboscis extension measured by recording the M17 muscle (% M17 (PER); *y*-axis) over successive conditioning trial (*x*-axis) shows that bees reliably learned to differentiate between a rewarded (CS+) and an unrewarded (CS-) light stimulus resulting in a stable memory (MEM) tested 10 min after the last acquisition trial (trial 10 and MEM: Chi2-test; *p* < 0.001). **(B)** During acquisition, response latency decreased (orange line, ±SD in grey). The median response time during memory test was ca. 2.9 s.

**FIGURE 4:**
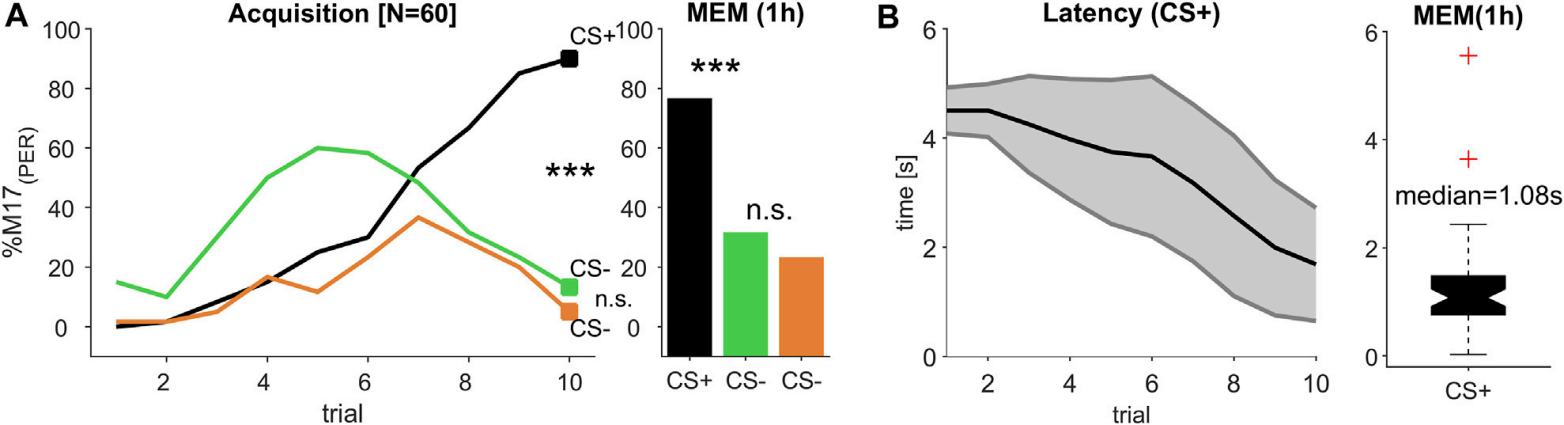
(Experiment 3): Decreasing response latency during multi-modal learning. **(A)** The percentage of bees responding with proboscis extension measured by recording the M17 muscle (% M17 (PER); *y*-axis) over successive conditioning trial (*x*-axis) shows that bees reliably learned to differentiate between a rewarded olfactory-visual compound stimulus (CS+) and its single unrewarded elements (CS-) odour (green) and light (orange), resulting in a stable memory (MEM) tested 1 h after the last acquisition trial (trial 10 and MEM: Chi2-test; *p* < 0.0003; significant after Bonferroni correction). **(B)** During acquisition, response latency decreased (black line, ±SD in grey). The median response time during memory test was ca. 1.08 s.

**FIGURE 5:**
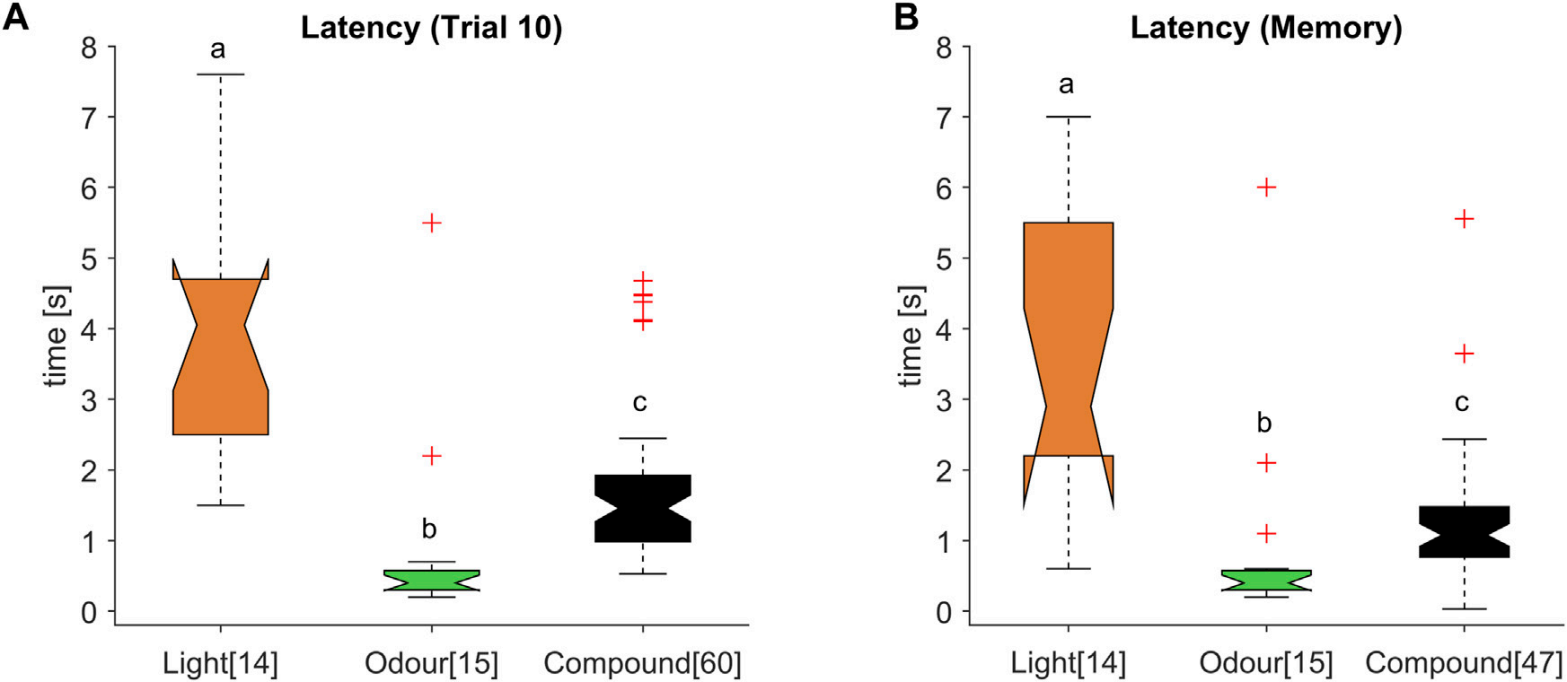
Intermediate response latencies for the olfactory-visual compound stimulus. **(A)** Response latency distribution for the reward associated stimulus during the 10th acquisition trial and **(B)** memory test were significantly different (ANOVA *p* < 0.001; *post hoc* Wilcoxon rank sum test: different letters indicate significant differences after Bonferroni correction; numbers in brackets refer to the number of tested animals). The multimodal compound (black) exhibited a decreased latency compared to the light stimulus (orange), whereas the latency was significantly higher compared to the odour stimulus (green).

To compare CS- and CS + responses, we used a Chi^2^-test for the 10th trial and the memory test. To account for multiple comparisons in the multimodal conditioning experiments, were one CS+ and two CS- stimuli (three stimuli in total) were compared (Figure 2; Figure 3; Figure 4), we adjusted the alpha-level to *p* < 0.016 (0.05/3) after Bonferroni’s correction. The different latency distributions of the three conditions were compared using an ANOVA (*p* < 0.05 followed by *post hoc* Wilcoxon rank sum test with an adjusted alpha-level of *p* < 0.016 (0.05/3) to account for multiple comparisons after Bonferroni’s correction (Figure 5).

## 3 Results

### 3.1 Response latency decreased during odour learning

Since the different odour combinations were learned equally well (Supplementary Figure S1), we combined both data sets, resulting in a total of 16 animals. During the acquisition phase, bees learned to significantly differentiate between the rewarded (CS+) and the unrewarded (CS-) odour stimulus, statistically tested at the last conditioning trial (trial 10; Chi^2^-test; *p* < 0.001, Figure 2A) and could correctly recall that valence 1 hour later during a memory test (MEM; Chi^2^-test; *p* < 0.001, Figure 2A). In the course of the learning phase, the M17 response to the CS + became faster and finally reached a response latency of about 400 m (median) during the memory test (Figure 2B). This value is in the range of odour processing speed after operant conditioning (Ditzen et al., 2003) and classical conditioning (Strube-Bloss et al., 2011) reported in earlier studies.

### 3.2 Response latency decreased during visual learning

Since both of the tested light combinations were learned equally well (Supplementary Figure S1), we combined the data of these experiments resulting in a total of 16 tested animals. During acquisition, bees learned to differentiate significantly between the rewarded (CS+) and the unrewarded (CS-) light stimulus (trial 10; Chi2-test; *p* < 0.001, Figure 3A) and remembered this stimulus valence also during a memory test (MEM; Chi2-test; *p* < 0.001, Figure 3A). Similar to the odour learning experiment, the time between stimulus onset and the activation of the M17 shortened with each trial (Figure 3B). However, in comparison to the response latencies observed during odour conditioning (Figure 2B), response time was much slower and reached 2.9 s (median) during the memory test (Figure 3B). Thus, although the acquisition pattern was similar during odour and visual learning (Figure 2A; Figure 3A), the time from stimulus onset to the final proboscis extension seemed strongly delayed for the light stimulus.

### 3.3 Acquisition time was prolonged during multimodal learning

We tested all combinations of two odour and two light stimuli in a classical positive patterning experiment, in which the single elements light and odour were presented unrewarded (CS-) and their combination in an olfactory-visual compound was presented rewarded (CS+). Since all four combinations elicited a reliable discrimination between the CS- elements and their multimodal compound (CS+), which represents an olfactory-visual reward association (Supplementary Figure S2), we merged data of all CS+ and each CS- element (Figure 4A), resulting in an absolute number of 60 tested bees. To establish a reliable multimodal reward association, which can be discriminated from its single elements, at least eight acquisition trials of each CS kind were needed. Compared to unimodal discrimination learning, were one to two trials (olfaction, Figure 2) or three to four trials (light, Figure 3) were sufficient to reach a high level of correct responses, the complexity of the multimodal learning task caused a higher rate of false positives to the single elements, which seemed to be dominated by the odour element (Figure 4A, trial 1–5). From trial 7 onwards, however, both single elements were discriminated from their compound, which was highly significant at the last acquisition trial (trial 10: Chi^2^-test; *p* < 0.0003; after Bonferroni correction), resulting in a stable and significant discrimination after 1 h consolidation time (MEM: Chi^2^-test; *p* < 0.0003; after Bonferroni correction).

### 3.4 Response latency decreased during multimodal learning

Similar to the unimodal learning (Figure 2; Figure 3) the response latencies showed a continuous decline over subsequent trials, indicating that the decision process to extend the proboscis accelerated with each trial (Figure 4B), resulting in a mean latency of 1.69 s (SD ± 1.04 s) at the 10th conditioning trial. Compared to the response latency, which was reached at the last conditioning trial during odour conditioning (mean = 0.85 s; SD ± 1.38 s), the processing of and responding to the CS + took about 0.84 s longer and was obviously delayed. However, when comparing the response latency of the last olfactory-visual CS + acquisition trial to the last visual CS + conditioning trial (mean = 3.86 s; SD ± 1.61 s), triggering the response behaviour (PER) was accelerated by about 2.17 s. Thus, the olfactory-visual compound seemed to be processed faster than the pure light stimulus, but slower than the pure odour stimulus.

### 3.5 Multimodal compound accelerates light but slows down odour perception

In all three experiments, the learning performance for the reward-associated stimuli (CS+) reached about 80%–90% at the last conditioning trial and were significantly different from the responses towards the unrewarded (CS-) stimuli. Moreover, the stimulus values were correctly remembered in the memory tests of all experiments (Figure 2A; Figure 3A and Figure 4A). We therefore compared the M17 response latency distributions between the three CS + stimuli (light, odour and compound) for the last conditioning trial (Figure 5A) and the memory test (Figure 5B). The medians for the last conditioning trial (Figure 5A) were 4.0 s for light, 0.4 s for odour and 1.4 s for the olfactory-visual compound stimulus. Almost the same values were observed during the memory test (light: median = 2.9 s; odour: median = 0.4 s; compound: median = 1.1 s). The difference of the M17 response latencies among the three experimental groups was significant for the last conditioning trial (Figure 5A; ANOVA *p* < 0.001, *post hoc* Wilcoxon rank sum test; Bonferroni correction, *p* < 0.0003) as well as for the memory test (Figure 5B; ANOVA *p* < 0.001, *post hoc* Wilcoxon rank sum test; Bonferroni correction, *p* < 0.0003). Thereby, the slowest response behaviour of the proboscis was triggered by the visual stimulus and the fastest PER by the odour stimulus. Most strikingly, the PER elicited by the olfactory-visual compound stimulus was located intermediate, between the unimodal stimuli. Thus, our results indicate that the olfactory-visual compound is processed faster than the light stimulus, and therefore, accelerates the unimodal light response. However, as the compound response is processed slower than the odour stimulus, it follows that it also slows down the unimodal odour response.

## 4 Discussion

### 4.1 Prolonged response time after visual conditioning

To precisely monitor the response latency during classical differential conditioning in which animals had to discriminate between two odour stimuli (experiment 1), two light stimuli (experiment 2) and an olfactory-visual compound stimulus and its single elements (odour and light; experiment 3), we recorded a myogram of the muscle M17, which is activating the proboscis. The M17 muscle on each hemisphere connects the ligular arm of the proboscis with the inner wall of the head capsule exoskeleton, where it is attached dorsally between the compound eye and the outer ocelli (Snodgrass, 1956). Such recordings were successfully used to analyse temporal aspects of odour discrimination (Smith and Menzel, 1989a; Smith and Menzel, 1989b) and reward expectation (Gil et al., 2008). Here we extended the scope of application to better understand modality-specific response latencies after classical conditioning of uni- and multi-modal stimuli. To ensure that the animals establish an olfactory-visual reward association we largely followed the CS-US design for multimodal positive patterning (PP) experiments introduced by Mansur and others (2018). Since this procedure includes 10 more CS- trials, the probability to receive a reward decreased from 50% during unimodal learning experiments (10 x CS+, 10 x CS-) to only 33% (10 x multimodal CS+, 10 x CS- light, 10 x CS- odour). We therefore shortened the ITI to 5 min to keep the animals motivated. Since bees were able to successfully solve multimodal PP experiments with even shorter ITIs (Mansur et al., 2018) we were certain that the animals can build a solid olfactory-visual reward association.

As already observed for odours (Figure 2; see also Smith and Menzel, 1989b), the conditioning of a light stimulus also led to a decreased M17 response latency in the course of training (Figure 3). Whereas odour learning resulted in a median latency of about 0.4 s during the memory test (Figure 2), which is in line with previous studies (Smith and Menzel, 1989a; Smith and Menzel, 1989b; Gil et al., 2008; Strube-Bloss et al., 2011), visually induced response latencies were found to be much longer (2.9 s median in the memory test, Figure 3B). Until recently it was assumed that visual conditioning in restrained honeybees is only possible, if either the antennae of the bees were cut off completely (Hori et al., 2006) or at least the flagellar segments at the tip were removed (Niggebrügge et al., 2009) to force the bees to learn the visual modality. However, in more recent studies it was proven that bees with intact antennae can also be trained to light stimuli (Dobrin and Fahrbach, 2012; Lichtenstein et al., 2018; Lichtenstein et al., 2019). Furthermore, it was shown that the length of the conditioned stimulus seemed to play a critical role, and that the light has to be presented at least 7 s in total with 3 seconds of overlap with the reward to be successfully learned (Lichtenstein et al., 2018). Our experimental stimulation design followed these rules (Figure 1), which resulted in a reliable acquisition in all visual discrimination tasks (Supplementary Figure S1). We were therefore certain that the measured response latencies reflect a realistic difference in computing a visual reward association compared to an olfactory one. Moreover, our findings were supported by visual observations of PER activity in bumblebees, showing latencies of about 3 s in response to an unimodal colour stimulus after absolute conditioning (Riveros, 2023).

### 4.2 Intermediate response time during olfactory-visual compound conditioning

As with olfactory and visual stimuli (Figure 2; Figure 3), the conditioning of an olfactory-visual compound stimulus resulted in a decreasing response latency during the acquisition phase (Figure 4), indicating that the animals become faster to evaluate the rewarded stimulus correctly and extend their proboscis in expectation of the reward. The response time reaches a median of 1.4 s during the last conditioning trial (Figure 5A) and was almost stable 1 hour later during the memory test (median = 1.1 s; Figure 5B). During compound stimulation both modalities were presented for 7 s in a completely overlapping manner. In a recent study, in which such a compound stimulus was used in a conditioning experiment in bumblebees (Riveros, 2023), the author measured response latencies of ca. 2 s on average, which was only about 0.5 s slower compared to our findings in honeybees. This slight discrepancy might be related to the different methods used. Whereas we precisely recorded the muscle potentials activating the PER, Riveros (2023) visually observed the PER every 0.5 s triggered by a metronome sound. However, the study supports our findings, since the unimodal visual response time (∼3–4 s in both species) appears to be delayed by about 1–2 s in honeybees (Figure 3) and bumblebees (Riveros, 2023) compared to the olfactory-visual compound. Thus, our data support the efficacy-based hypotheses (Kulahci et al., 2008), namely, that multimodal stimuli enhance decision making, resulting in faster behavioural responses. On the other hand, when comparing our data to an olfactory stimulus, we found the opposite effect. In this case, the olfactory-visual compound stimulus delayed the response time, which might be the result of prolonged processing of the multimodal olfactory-visual reward association compared to a unimodal odour reward association. Although it seems disadvantageous for the animal at a first glance, such increased processing/sampling time was reported to improve the ability to recognise and differentiate low and very low odour concentrations (Wright et al., 2009) and thus increased the reliability of correct behavioural responses. Taken together, our results provide evidence for the basic assumption of multimodal integration, namely, the interaction of single modalities of a multimodal compound with each other by weakening and/or enhancing the processing of these single modalities (Calvert et al., 2004). In our case the response time to the olfactory modality was decelerated whereas the behavioural response time to the visual modality was accelerated compared to response time induced by the multimodal olfactory-visual compound stimulus (Figure 5).

### 4.3 Olfactory-visual convergence in the bee brain

Multimodal integration involves neural convergence of different sensory pathways. Olfactory projection neurons of the antennal lobes (AL) as well as visual projection neurons of the optic lobes innervate the calyces of the mushroom bodies (MB), where they converge on about 180.000 Kenyon cells, the MB principal neurons (for review see: (Fahrbach, 2006; Menzel, 2014; Groh and Rössler, 2020)). The information is further converged to a group of approximately 400 mushroom body output neurons (MBON) with long-range centrifugal connections to the lateral horn, protocerebral lobe, AL and to the contralateral brain side, and close-range feedback connections to the MB calyx (Rybak and Menzel, 1993; Strausfeld, 2002). Recent experiments have shown that a MBON subpopulation, which account for about 32% in the related study, respond to both visual and olfactory stimuli (Strube-Bloss and Rössler, 2018). The population activity in these multimodal MBONs separates olfactory from visual information, as well as unimodal information from their olfactory-visual compound, and thus is able to categorise incoming stimuli. An additional category of visual stimuli was shown to be computed by another subpopulation of MBONs, which separates UV-light from other visual stimuli (Schmalz et al., 2022).

Classical differential odour conditioning recruits insensitive MBONs to code for the reward-associated stimulus (Strube-Bloss et al., 2011; 2016). Therefore, the reward association can be a combination of complex stimulus features including odour identity and stimulation side encoded in the recruited MBON population activity. For example, an odour conditioned to the left antennae evoked a different activity pattern compared to the same odour presented to the right antennae (Strube-Bloss et al., 2016). Thus, the activity of recruited MBON integrates different stimulus features to a new representation of the reward associated compound stimulus. Such a compound was learned in our experiment 3 (Figure 4), in which the animals had to differentiate the single elements (odour and light) from their rewarded olfactory-visual compound. In this respect, it is most likely that our conditioning procedure was accompanied by the recruitment of initially non responding MBONs (ca. 17% of the population; (Strube-Bloss and Rössler, 2018)), which code after learning for the olfactory-visual compound. These MBONs may encode the behavioural decisions during sensory-motor transformation, as it was shown for MBONs in untrained cockroaches (Arican et al., 2022). In addition to a previously studied group of GABAergic MBONs, forming recurrent circuits within the MBs (Grünewald, 1999a; 1999b; Haehnel and Menzel, 2010; 2012), anatomical characterisation revealed MBONs with potential top-down recurrent connections to the AL (Rybak and Menzel, 1993; Kirschner et al., 2006). If we assume that such centrifugal-feedback neurons were recruited during the olfactory-visual conditioning they may allow the visual modality to influence odour processing at the AL-level, and might be responsible for the delayed response time we measured. Such cross-modal modulation of olfaction has been shown in the moth *Spodoptera littoralis,* in which the predator sound of a bat induced long-term sensitization of olfactory neurons in the AL (Anton et al., 2011). However, multimodal feedback might not be involved during olfactory learning, which might be the reason for the fast response time we observed. On the other hand, a similar type of centrifugal feedback connectivity like described above, might exist connecting the MB output with the optic lobes. In that case, the olfactory modality might modulate the visual processing and speed up its computation, resulting in a faster response compared to the unimodal visually triggered response. Knowledge about the influence and contributions of different MBON subpopulations will be addressed in future experiments, in which we will combine olfactory-visual compound conditioning with extracellular long term recordings of MBONs.

## Data Availability

The original contributions presented in the study are included in the article/Supplementary Material, further inquiries can be directed to the corresponding author.
