# Supplementary material for: Visual accelerated and olfactory decelerated responses during multimodal learning in honeybees
Source: Front Physiol. 2023 Oct 20;14:1257465. doi: 10.3389/fphys.2023.1257465 (PMC10624174; doi:10.3389/fphys.2023.1257465)
Supplement: Supplementary file 1 [file Presentation1.pdf]

Supplementary Material

Visual accelerated and olfactory decelerated responses during multimodal learning in honeybees

Martin Strube-Bloss[1*], Patrick Günzel[1], Carmen A. Nebauer[2,3] and Johannes Spaethe[3]

[1*]Department of Biological Cybernetics, Faculty of Biology, Bielefeld University, Universitaetsstr. 25, 33615 Bielefeld, Germany

[2] Department of Plant-Insect-Interaction, Life Science Systems, Technical University of Munich, Hans-Carl-von-Carlowitz-Platz 2, 85354 Freising-Weihenstephan, Germany

[3]Behavioral Physiology and Sociobiology (Zoology II), Biocenter, University of Würzburg, Am Hubland, 97074 Würzburg, Germany

***corresponding author: phone:** 0521 106-5532 / **e-mail:** martin.strube-bloss@uni-bielefeld.de

Supplementary Figures (Experiment 1 and 2)

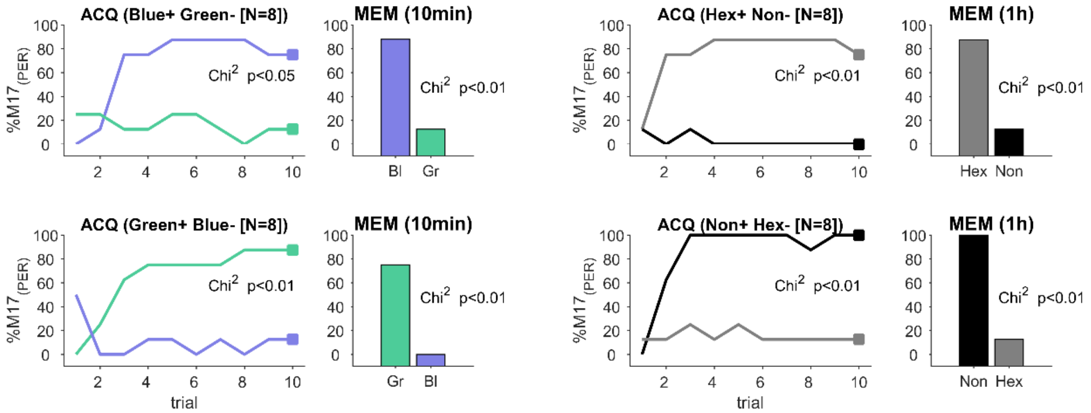

Figure S1: Single element conditioning. (Left) Two light stimuli were used and differentially conditioned to 16 bees in total (Experiment 1). Half of them learned that blue was presented rewarded and green unrewarded (upper panels). The other half learned the opposite meaning (lower panels). In both cases animals significantly learned to discriminate between the two lights (trial 10 and MEM: Chi^2-test; $p<0.05$). **(Right)** Two odour stimuli were used and differentially conditioned to 16 bees in total (Experiment 2). Half of them learned that Hexanol was presented rewarded and Nonanol unrewarded (upper panels). The other half learned the

opposite meaning (lower panels). In both cases animals significantly learned to discriminate between the two odors (trial 10 and MEM: Chi^2-test; $p<0.01$).

Supplementary Figures (Experiment 3)

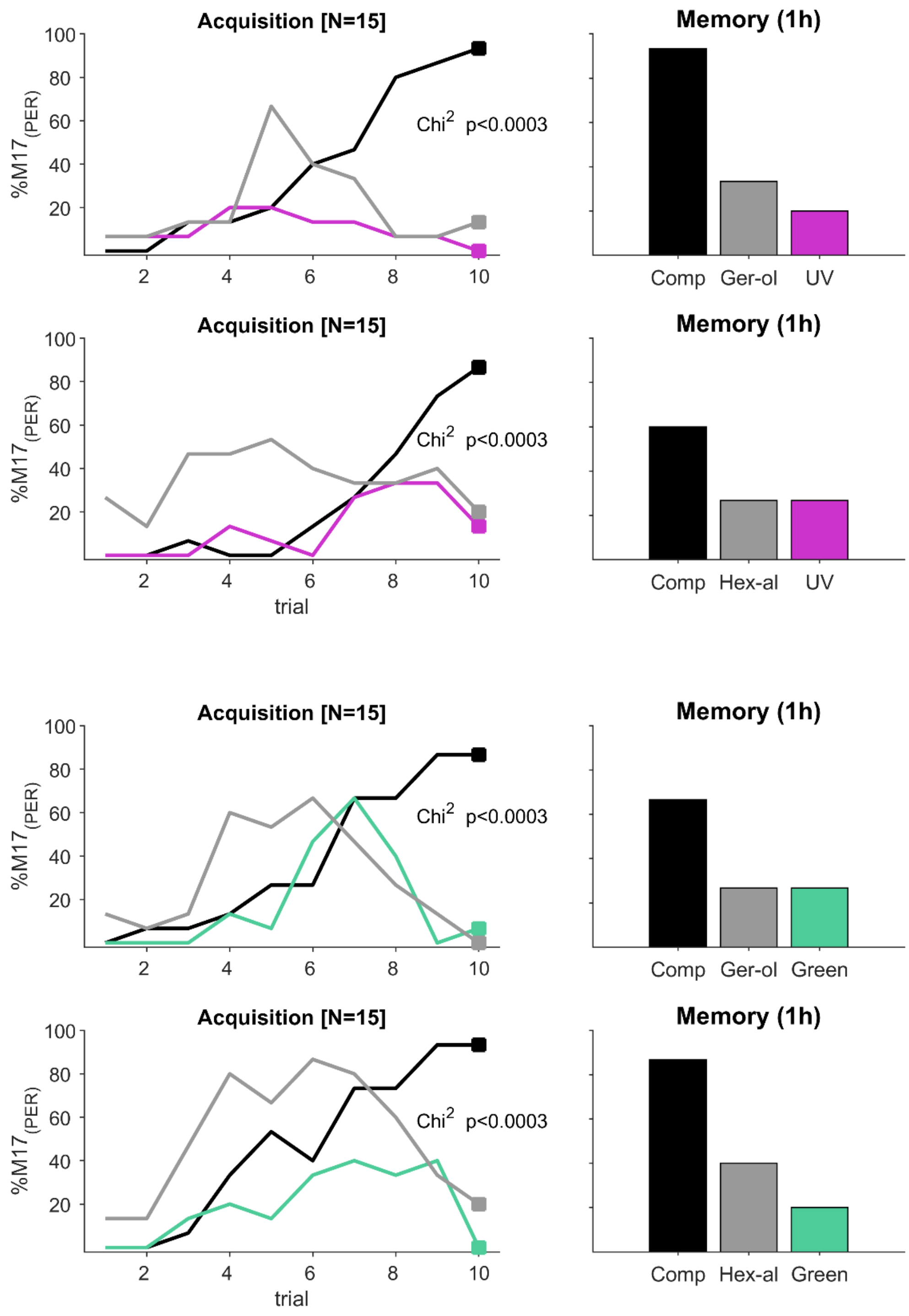

Figure S2: Compound conditioning of 60 bees (Experiment 3). The compound of UV-light and Geraniol was presented rewarded (CS+), whereas the single elements were presented unrewarded (CS-). One hour after the last conditioning trial both CS- as well as the CS+ was tested for memory in a randomized fashion (first row; N=15). To ensure, that the odour has no

influence on the discrimination we repeated the experiment by replacing Geraniol by Hexanal (second row; N=15). In both cases bees significantly learned to differentiate the compound from its single elements (trial 10 and Memory: Chi^2-test; $p<0.0003$). To make sure, that the identity of the light has no influence on the discrimination skills we repeated the experiments by replacing UV-light by green (row 3 and 4). Each odour-light compound was significantly separated from its single unrewarded elements ((trial 10 and Memory: Chi^2-test; $p<0.0003$)
